# Community-Based Organizations as Effective Partners in the Battle Against Misinformation

**DOI:** 10.3389/fpubh.2022.853736

**Published:** 2022-03-15

**Authors:** Maya Rom Korin, Faven Araya, Muhammed Yassin Idris, Humberto Brown, Luz Claudio

**Affiliations:** ^1^Icahn School of Medicine at Mount Sinai, New York, NY, United States; ^2^Arthur Ashe Institute for Urban Health, Brooklyn, NY, United States; ^3^Morehouse School of Medicine, Atlanta, GA, United States

**Keywords:** misinformation, community-based organizations, health literacy, social media, community health, health communication

## Introduction

The COVID-19 pandemic has increased the need for delivering accurate and timely health information to the public (1). However, the public is being increasingly exposed to a barrage of health misinformation amplified by social media (2–4). The World Health Organization (WHO) and the United Nations coined the term “infodemic” to describe this unprecedented spread of health misinformation (5). A recent report by the United States Surgeon General's Advisory highlighted how the rapid proliferation and decentralization of health information coupled with insufficient communication from trusted sources has led to the public's increased exposure to health misinformation (6). Health misinformation easily spreads in the current communications environment that includes social media, independent news outlets, and online forums that feed content into algorithms which often prioritize popularity and controversy over accuracy (4, 6, 7).

Misinformation is more likely to take hold when people have poor eHealth literacy and thus are unable to appraise health information (2, 8–10). While health literacy is broadly defined as the skills needed to make health decisions in the context of everyday life, eHealth literacy is “the ability to seek, find, understand, and appraise health information from electronic sources and apply the knowledge gained to addressing or solving a health problem” (9). Having eHealth literacy is essential for individuals to be able to wade through the myriad of information that is found online, particularly in a highly politicized environment where there is a vacuum of credible and trusted sources of information (10–12). It is important to note that eHealth literacy is not equally distributed. Social determinants of health shape the accessibility to and use of information channels and the ability to process health information, the comprehension of health information, and the capacity to act upon that knowledge (13–17). Additionally, it is estimated that over 40 million adults in the United States have low literacy skills, resulting in health disparities and limiting equitable access to health resources (18, 19). The combination of poor health literacy and poor ehealth literacy allows misinformation to take hold (9, 11, 20).

While the US Surgeon General has called upon health organizations to partner with community members to develop and disseminate health messaging, the potential contribution of community-based organizations (CBOs) as trusted conduits is being missed (6). CBOs are essential health stakeholders who have established relationships with communities that are often overlooked by the larger healthcare system (21). We argue that including CBOs early in the health communication pathway is critically needed to combat this infodemic and reorient communities to their already trusted sources of health information. CBOs have tremendous reach within the communities that they serve, providing social networking, encouraging health promoting behaviors, and implementing health interventions through multiple modalities of community engagement (22). Additionally, because true community engagement and not simply community outreach is needed to gain the trust of marginalized populations, CBOs have a distinct advantage as they are already embedded within the fabric of the community.

## Community Based Organizations: Integral for the Health Communication Cycle

CBOs can be exceptionally effective in health communication and health promotion planning because they are rich with social capital (22–24). Having social capital uniquely positions CBOs to identify the social networks and normative behavior within a community, particularly during a public health emergency (25, 26). This understanding is essential to implement an effective health communication strategy and combating misinformation.

The Health Communication Cycle typically involves four phases: Planning, Developing, Implementing, and Evaluating (27). While in theory the Health Communication Cycle encourages involving the community in testing health communication materials and helping in the dissemination phase (28), the role of CBOs is usually limited to community outreach. Instead of this, we recommend that CBOs be closely integrated even before the four stages of the health communication process. A first step, before planning a health communication project, government or academic health agencies should identify CBOs that are well-integrated in the communities to be served. Public health workers should approach CBOs that are already working in health-related issues that affect the relevant populations. The focus of their programming and services should already be serving the population that is of interest for the research study or intervention, thus the work should fit seamlessly into their interests. Importantly, partnerships with CBOs should not be done in a *post-hoc* fashion but should exist before health communication needs arise.

Once partner CBOs have been identified and clear collaborative common goals have been established, then it is important to integrate CBOs into the Planning Phase of the project. Their early involvement in the planning phase should include analyzing the problem, setting the intervention strategy, deciding on the population to be served, and co-creating health communication content. Partnering CBOs are needed to properly identify the health concerns of the community, so that ineffective content is not developed that shows additional mistrust in the source. Planning efforts need to be collaborative from the beginning, from setting an initial agenda to co-ownership of all health communication materials and tools created. CBOs should be involved in creating the agenda and all aspects of planning instead of being used to approve a preliminary plan. Our recommendation is that Planning Phase meetings with CBOs should start on a blank page onto which CBOs and public health workers have equal say from the very beginning of the process.

In the Development Phase of the Health Communication Cycle, CBOs expertise and nuanced understanding of their constituents ensures that information is created in a way that meets the health literacy needs of the communities and that is culturally appropriate. Recent communications around vaccination often failed to reach communities that were impacted most by COVID-19 because the messages developed lacked cultural sensitivity, linguistic nuance, and the involvement of trusted messengers. For example, messaging created by local health authorities that targeted the Afro-Caribbean community in Brooklyn was not appropriately translated and did not consider the diversity within this unique community. This communication campaign led to fragmented efforts that failed to reach the communities most in need of the information.

The Implementation Phase of the Health Communication Cycle involves preparing and distributing information to the population to be served. The role of CBOs in this phase can include disseminating health information through the appropriate existing channels. In this rapidly changing digital landscape, communities need to be reached in whatever medium is already most accessible to them, whether that be text messaging, email, or social media platforms. CBOs have a distinct advantage, as they are equipped with local knowledge, expertise, and trusted relationships to determine the best means of communication with their constituents leading to more efficacious health communication strategies (22, 29, 30). As such, the partnership should include community leaders that are seen as trusted messengers. For example, recognizing a unique relationship amongst members of their community, Arthur Ashe Institute for Urban Health partners with barbers and hair stylists to relay health related messages around COVID-19 to their patrons. This kind of health communication dissemination can only take place if well-established CBOs are included in all aspects of health communication efforts. Additionally, co-ownership of health communication materials ensures that CBOs have the resources, interest, and investment to adequately address the established common goal.

Finally, in the Evaluation Phase, CBOs should have a connection with community members that allows for a productive feedback loop to evaluate the effectiveness of the communication and identify changing community concerns to inform future messaging. CBOs bring the perspective of the on-the-ground experiences that inform appropriate evaluation measures/metrics that capture the health communication interventions scope, reach and effectiveness (22, 31). Elements of evaluation and measures of success need to be co-defined with CBOs during the Planning Phase of the project. This is important, as health authorities and academic researchers may have different ideas of what constitutes a successful communication campaign. For example, scientific publication may be an important metric of success, especially for academics. If scientific publication is a goal, it is important that partner CBOs participate in the authorship process. In the instance of the current publication, both the academics and community leaders shared responsibility and co-authorship.

## Conclusion

In the dynamic fast-changing pandemic environment we currently inhabit, misinformation has real-world effects on population health. The traditional means of health communication placed trusted intermediaries between information creating sources and information receivers. Our current communication landscape removes these intermediaries, allowing for misinformation to proliferate. Part of the problem has been that health information created and disseminated by public health organizations is not often tailored to the needs of those at highest risk, deepening gaps in health disparities and furthering mistrust and skepticism.

While public health authorities sometimes engage CBOs for community outreach, they miss opportunities to leverage the inroads of trust that CBOs have formed in their communities to meaningfully engage in all phases of the Health Communication Cycle. Understanding what information is needed, creating messaging that is appropriate and relevant, and disseminating information in whatever means works best are essential steps in battling the infodemic. CBOs are trusted entities that are deeply embedded within the communities they serve and have a nuanced understanding of their constituents that is essential to combating misinformation. Trust, a fundamental principle in relationship building, is a unique and intangible factor at the core of CBOs that positions them well to play an active role in the health communication cycle fostering health equity and promoting equal opportunity to health. Thus, to effectively communicate and fight against health misinformation, particularly in populations with deep-seated mistrust or poor health literacy, we must include and engage CBOs in all facets of health communication.

## Author Contributions

MK led the writing of the manuscript. MI, LC, and FA contributed both in writing and editorial feedback. HB provided editorial feedback. All authors contributed to the article and approved the submitted version.

## Funding

This work was supported by the National Institutes of Health (P30-023515 and R25 HL108857-06).

## Conflict of Interest

The authors declare that the research was conducted in the absence of any commercial or financial relationships that could be construed as a potential conflict of interest.

## Publisher's Note

All claims expressed in this article are solely those of the authors and do not necessarily represent those of their affiliated organizations, or those of the publisher, the editors and the reviewers. Any product that may be evaluated in this article, or claim that may be made by its manufacturer, is not guaranteed or endorsed by the publisher.
